# High-frame-rate contrast-enhanced ultrasound to differentiate between clear cell renal cell carcinoma and angiomyolipoma

**DOI:** 10.1186/s12885-024-12413-4

**Published:** 2024-05-30

**Authors:** JingLing Wang, JiaYu Shi, Long Gao, WeiHong Hu, Miao Chen, WeiPing Zhang

**Affiliations:** 1 Department of Ultrasound, The First Affiliated Hospital, Jiangxi Medical College, Nanchang University, Nanchang, China; 2 The First Clinical Medical College, Jiangxi Medical College, Nanchang University, Nanchang, China; 3 School of Advanced Manufacturing, Nanchang University, Nanchang, China

**Keywords:** High-frame-rate, Contrast enhanced ultrasound, Clear cell renal cell carcinoma, Angiomyolipoma, Differential diagnosis

## Abstract

**Background:**

To investigate the diagnostic efficacy of high-frame-rate contrast-enhanced ultrasound (H-CEUS) in differentiating between clear cell renal cell carcinoma (CCRCC) and angiomyolipoma (AML).

**Methods:**

A retrospective study was performed on the clinical data of 79 patients diagnosed with CCRCC and 31 patients diagnosed with AML at the First Affiliated Hospital of Nanchang University between October 2022 and December 2023. Conventional ultrasound (US) and H-CEUS examinations were conducted on all patients prior to surgery, dynamic images were recorded from the US, and the qualitative and quantitative parameters of H-CEUS were collected. The t-test, χ² test and non-parametric Mann-Whitney test were employed to assess differences in clinical data, US characteristics, and qualitative and quantitative parameters of H-CEUS between the CCRCC and AML groups. The independent risk factors of CCRCC were identified using binary logistic regression. The receiver operator characteristic (ROC) curve was constructed to evaluate the diagnostic effectiveness of clinical + US and H-CEUS in differentiating between CCRCC and AML.

**Results:**

The CCRCC group and the AML group exhibited significant differences in patient gender, operation mode, nodular echo, and nodule blood flow (χ²=11.698, -, -,=10.582; *P*<0.001, <0.001, <0.001, and = 0.014, respectively). In addition, the H-CEUS qualitative analysis demonstrated significant differences between the AML group and the CCRCC group with respect to enhancement mode, regression mode, peak intensity, enhancement uniformity, no enhancement, and presence or absence of pseudocapsule (χ²=41.614, -, -, = 2.758, = 42.099, -; *P*<0.001, <0.001, <0.001, 0.097, <0.001, and <0.001, respectively). The Arrival time (AT) in the CCRCC group was significantly shorter than that in the AML group, as determined by quantitative analysis of H-CEUS (Z=-3.266, *P* = 0.001). Furthermore, the Peak intensity (PI), Ascent slope (AS), and The area under the curve (AUC) exhibited significantly higher values in the CCRCC group compared to the AML group (Z=-2.043,=-2.545,=-3.565; *P* = 0.041, = 0.011, and <0.001, respectively). Logistic regression analysis indicated that only gender, nodule echo, the pseudocapsule, AS, and AUC of H-CEUS were independent risk factors of CCRCC. The ROC curve revealed that combining gender and nodule echo yielded a sensitivity of 92.4%, specificity of 64.5%, and an AUC of 0.847 in distinguishing between CCRCC and AML. When combining the H-CEUS parameters of pseudocapsule, AS, and AUC, the sensitivity, specificity, and AUC for distinguishing between CCRCC and AML were 84.8%, 96.8%, and 0.918, respectively. No statistically significant difference was observed in the diagnostic effectiveness of the two methods (Z=-1.286, *P* = 0.198). However, H-CEUS demonstrated better AUC and specificity.

**Conclusions:**

H-CEUS enhances the sensitivity and specificity of differentiating between CCRCC and AML by improving the temporal resolution, offering a more precise diagnostic foundation for identifying the most appropriate therapy for patients.

## Background

Clear cell renal cell carcinoma (CCRCC) accounts for about 90% of all renal malignancies [1]. Renal cell carcinoma (RCC) has a 2–3 times higher prevalence in men than in women, with an average age of onset of approximately 65 years [2]. In clinical practice, angiomyolipoma (AML) is widely recognized as the most prevalent benign solid kidney tumor [3]. Imaging examinations play a crucial role in the diagnosis of auxiliary characteristics associated with CCRCC and renal AML, including tumor size, presence of metastasis, lymph node enlargement, extra-renal extension, renal pelvic involvement, and renal vein involvement. However, the treatment approaches for CCRCC and renal AML differ significantly. CCRCC is primarily treated by radical nephrectomy, whereas renal AML is typically observed via follow-up, or may be treated by minimally invasive procedures or radiofrequency ablation surgery. Therefore, differentiating between AML and CCRCC holds great clinical significance. Otherwise, an unclear diagnosis may subject AML patients to unneeded surgical procedures.

Computed tomography (CT) and magnetic resonance imaging (MRI) have notable sensitivity and specificity [4, 5] and are often employed for the clinical differential diagnosis of CCRCC and AML. However, their application is occasionally constrained by factors such as radiation exposure, nephrotoxicity, and the presence of metal implants. Contrast-enhanced ultrasound (CEUS) may be utilized to differentiate between malignant and benign lesions through the visualization of tumor blood vessels. The tumor type can be predicted using CEUS, which gathers information on multiple parameters such as enhancement mode, regression mode, peak intensity, enhancement direction, and enhancement form [6, 7]. CCRCC is often distinguished by early inhomogeneous intensification [8] and rapid regression. In addition, the enhanced margin encircling the lesion, referred to as a pseudocapsule, is most evident during the late stages of regression, which is a characteristic feature of the diagnosis of CCRCC [9]. In contrast, AML exhibits a lesser degree of enhancement compared to the neighboring renal cortex, indicating a modest level of enhancement [10]. However, in the field of clinical practice, CEUS lacks sufficient reliability for distinguishing between tumors, and significant overlap is found across various types of tissues. In particular, reliably distinguishing hypovascular AML from CCRCC remains challenging. Furthermore, despite being regarded as a real-time imaging technology, the current contrast image frame rate of CEUS has limitations. In cases with a small tumor volume and abundant blood supply, the arterial phase perfusion process occurs rapidly. Therefore, the vascular morphology may not be accurately depicted due to the low frame rate of CEUS. Consequently, this hampers the diagnostic efficiency.According to the literature [11], the blood vessels of the kidney are abundant, which is equivalent to 1/5 to 1/4 of the cardiac output. Whether the kidney tumor is benign or malignant, low frame frequency contrast-enhanced ultrasonography can show high enhancement of rich blood supply, which can easily lead to misdiagnosis.

The utilization of high-frame-rate CEUS (H-CEUS) imaging technology typically involves a limited number of emission events to achieve comprehensive imaging of a specific area. The emitted ultrasonic field is commonly chosen as a plane wave field, which facilitates the acquisition of image data within a rectangular area. This approach also results in an increased frame frequency of contrast, ranging from 50 to 80 Hz, surpassing the CEUS frame rate presently implemented in clinical settings (10–15 Hz). To a certain extent, the higher frame rate mitigates the limitations of the low frame rate on the temporal resolution of contrast images. Increasing the contrast frame rate could enhance the temporal resolution of contrast images, thereby enabling a clearer and more precise display of the contrast agent’s perfusion process, which may yield additional diagnostic insights [12].

This study investigated the differences in the contrast perfusion process of H-CEUS in CCRCC and AML using qualitative and quantitative analysis. Additionally, it evaluated the differential diagnostic effectiveness of clinical, US, and H-CEUS parameters for these two tumors using the receiver operating characteristic (ROC) curve. The objective was to explore the potential value of H-CEUS in the differential diagnosis of CCRCC and AML.

## Methods

### Patient information

From October 2022 to March 2024, a cohort of 110 consecutive patients with renal masses who underwent US and H-CEUS examinations at the First Affiliated Hospital of Nanchang University were chosen. Among these patients, 79 had CCRCC (with a mean age of 58.76 ± 11.79 years) and 31 had AML (with a mean age of 57.26 ± 7.09 years). The inclusion criteria in this study were as follows. (1) The gray-scale ultrasound clearly detected renal mass. (2) A postoperative pathological diagnosis of CCRCC was confirmed for all CCRCC patients. (3) Postoperative pathology or enhanced CT examination confirmed the presence of AML lesions, showing no significant change in the enhanced CT follow-up for a period of 3–6 months. (4) All H-CEUS examinations were performed; the time-intensity curve (TIC) parameters of the renal lesions were obtained, and the goodness of fit (GOF) of the lesion TIC curve > 0.7. The exclusion criteria included the following. (1) Unsatisfactory imaging quality of conventional gray-scale ultrasound and H-CEUS. (2) Contraindications to CEUS, such as CEUS allergy and lactating women. (3) Inadequate dynamic image storage leading to difficult analysis of TIC parameters. (4) CEUS images that could not be interpreted due to deep tumor location, patient obesity, or poor ultrasound penetration. This study was approved by the Medical Ethics Committee of our hospital (Clinical Trial Number: IIT2023166, Ethics: IIT2023174), and each patient provided informed consent.

### Equipment and examination techniques

#### Equipment

The Mindray Resona R9 color Doppler ultrasound diagnostic apparatus was utilized with the SC 5-1U probe, and operated at a frequency of 3–5 MHz. CEUS technology uses ultra-wideband nonlinear (UWN) signals. In conjunction with a low mechanical index of 0.06–0.08, the H-CEUS imaging procedure utilized an image frame rate between 50 and 65 Hz. The ultrasonic contrast agent (SonoVue, Bracco company, Italy) was mostly composed of sulfur hexafluoride microbubbles. A suspension was formed by vigorously mixing 5 ml of 0.9% sodium chloride solution with 59 mg of SonoVue before use.

#### Ultrasound and H-CEUS examination

Patients underwent conventional renal ultrasound and H-CEUS examination prior to surgery or enhanced CT. The US examination documented the lesion position, lesion quantity, lesion dimension, echo characteristics, and blood flow. Subsequently, the H-CEUS mode was employed to observe the long-axis section containing the lesion and the surrounding normal renal tissue. 1.0 ml of SonoVue suspension was administered through the superficial elbow vein, followed by 5 ml of a 0.9% sodium chloride solution for flushing. A series of dynamic photos were captured and retained for 5 min. In the absence of pathology or enhanced CT results, two sonographers with extensive expertise in CEUS (with a minimum of five years of experience in this field) conducted independent analyses of the images. Discrepancies in the two results were ascertained through collaborative discussion.

#### US image features

The following features were obtained from US images: (1) Nodule side (left/right); (2) Location of nodules (upper/middle/lower pole); (3) Nodular composition (solid/cystic); (4) Nodule echo (hyperechonic/hypoechoic/isoechoic); (5) Nodule boundary (clear/unclear); (6) Nodule morphology (regular/irregular); (7) Liquefaction state (present/absent; 8) Color Doppler blood flow imaging was employed to detect the tumor blood flow signal [13], which was categorized into four grades: grade 0 (no blood flow seen inside the tumor), grade I (a small amount of 1 to 2 star-shaped blood flows), grade II (moderate blood flow showing 3–4 star-shaped or short beam-like blood flow), and grade III (rich blood flow showing 2–3 or more colors blood flow, reticular or branched). 9) Calcification (present/absent).

#### Qualitative analysis of H-CEUS images

The renal angiography was divided into the perfusion phase (0–30 s) and the regression phase (> 30s), with a focus on observing the enhancement and regression modes, peak intensity, enhancement homogeneity, enhancement morphology, and annular enhancement of the mass. (1) Enhancement mode: the enhancement time in the lesion and that of the adjacent normal renal cortex were compared. Fast forward indicated an earlier lesion enhancement time compared to the renal cortex, slow forward indicated a later lesion enhancement, and equal forward suggested a similar lesion and renal cortex enhancement time. (2) Regression mode: rapid regression was characterized by the contrast agent clearance in the lesion surpassing that of the adjacent normal renal cortex. Slow regression was denoted by delayed contrast agent clearance in the lesion compared to the adjacent normal renal cortex. Similar contrast clearance between the lesion and the surrounding normal renal cortex indicated equal regression. (3) Peak intensity: a high level of enhancement indicated a higher peak intensity of the lesion enhancement compared to that of the surrounding normal renal cortex. Conversely, similar intensity indicated an equal level of enhancement. A lower intensity of the lesion enhancement compared to the surrounding normal renal cortex indicated a low level of enhancement. (4) Enhancement uniformity: The distribution of enhancement intensity inside the lesion was categorized into homogeneous and heterogeneous lesion enhancements. (5) No enhancement: No enhancement was defined as an absence of any increase in contrast observed in the lesion. (6) Enhancement direction: Centripetal enhancement referred to the enhancement of a lesion initiating from its periphery and progressing towards its center. Centrifugal enhancement, on the other hand, denoted the enhancement initiated from the center of the lesion and extended towards its periphery. Lastly, diffuse enhancement indicated the concurrent enhancement of the lesion’s periphery and center. (7) Boundary after enhancement: The boundary line between the lesion site and the adjacent normal renal cortex after contrast enhancement was categorized as either clear or unclear. (8) Enhancement range: The region of increased echo caused by the contrast agent in the lesion site and its adjacent normal renal cortex was divided into enlargement and no enlargement. (9) Pseudocapsule: The presence of a ring-shaped high enhancement surrounding the enhanced nodule may be classified into two categories: with a pseudocapsule and without a pseudocapsule.

#### Quantitative analysis of H-CEUS images

Mindray Resona 9 color Doppler ultrasound diagnostic instrument was used with the integrated analytic software to perform quantitative analysis of the TIC. The region of interest (ROI) was set and positioned within the kidney lesion. Notably, the ROI in the lesion area should avoid large blood vessels and necrotic areas. In cases with heterogeneous enhancement, the area with the highest enhancement intensity was recorded, while the ring enhancement area was avoided. The primary parameters of the study included the following. (1) GOF represented the degree of fit between the fitting curve and the original curve, ranging from 0 to 1. A value of 1 signified a perfect fit between the two curves. (2) Arrival time (AT) referred to the specific time when the contrast intensity first becomes visible. (3) Time to peak (TTP) referred to the time point when the contrast intensity reached its highest level. (4) Peak intensity (PI). (5) Ascent slope (AS) indicated the slope between the two points on the curve that represent the initial lesion perfusion and the peak. (6) 1/2 descending time (DT/2) referred to the time taken for the intensity to decline to half of the peak intensity after reaching its maximum. (7) Descending slope of curve (DS). (8) The area under the curve (AUC) was the area under the time-intensity curve of the contrast process. (9) The mean transit time (MTT) = DT/2 – AT.

### Statistical analysis

The statistical software SPSS 26.0 was utilized to analyze the data presented above. The age and maximum diameter of the nodule were expressed as x ± s, which conformed to a normal distribution. The t-test was employed to assess the differences between the CCRCC group and the AML group. Gender, nodule characteristics, and US characteristics were count data and were expressed as the number of cases. The χ² test or Fisher’s exact probability method was applied to compare the differences between the two groups. The enhanced feature of qualitative analysis of H-CEU was count data (the number of cases), and the comparison between the two groups was conducted using either χ² test or the Fisher exact probability method. Quantitative analysis of the TIC parameters of H-CEUS was expressed as M (QR), which deviated from a normal distribution. Consequently, non-parametric Mann-Whitney test was employed for comparisons between the two groups.In addition, independent risk factors associated with CCRCC were identified by logistic regression analysis and incorporated the indicators that exhibited statistically significant differences. The Z test was utilized to compare and contrast the diagnostic efficacy of clinical plus US and H-CEUS in distinguishing between CCRCC and AML, as determined by the ROC curve. *P* < 0.05 indicated a statistically significant difference.

## Results

### General state and US features

The comparison of general parameters and US between the CCRCC and AML groups is shown in Table 1. Gender, surgical method, nodule echo, and nodule blood flow demonstrated significant differences between the CCRCC group and the AML group (χ² = 11.698, -, -, = 10.582; *P* < 0.001, < 0.001, < 0.001, and = 0.014, respectively). The majority of the CCRCC group (62.0%) were men, with 34 cases (43%) who underwent radical nephrectomy and 45 cases who underwent partial nephrectomy. On the other hand, the AML group mostly consisted of women (74.2%), with 19 cases (61.3%) undergoing partial nephrectomy and 12 cases (38.7%) under follow-up surveillance. US of CCRCC was mainly characterized by hypoechoic lesions (68.4%) with grade II and III blood flow within the nodule (53.2%); in contrast, AML was mostly defined by hyperechoic lesions (83.9%) with blood flow grades 0 and I within the nodule (77.4%).

**Table 1:** Characteristics of Clinical parameter and conventional US in CCRCC and AML groups

		CCRCC(*n* = 79)	AML(*n* = 31)	χ²or t	*p* Value
Gender, n(%)	Male	49(62.0%)	8(25.8%)	11.698	< 0.001
	Female	30(38.0%)	23(74.2%)		
Age(years): mean ± STD		58.76 ± 11.79	57.26 ± 7.09	0.662	0.509
Laterality, n(%)	Left	48(60.8%)	12(38.7%)	4.366	0.055
	Right	31(39.2%)	19(39.2%)		
Location, n(%)	Superior	30(38.0%)	12(38.7%)	2.252	0.324
	Middle	28(35.4%)	7(22.6%)		
	Inferior	21(26.6%)	12(38.7%)		
Surgery, n(%)	Radical nephrectomy	34(43.0%)	0(0.0%)	-	< 0.001
	Partial nephrectomy	45(57.0%)	19(61.3%)		
	Unoperated	0(0.0%)	12(38.7%)		
Echogenicity, (n/%)	Hypo-	54(68.4%)	5(16.1%)	-	< 0.001
	Iso-	2(2.5%)	0(0.0%)		
	Hyper-	23(29.1%)	26(83.9%)		
Boundary, n(%)	Well defined	77(97.5%)	30(96.8%)	-	1.000
	Poorly defined	2(2.5%)	1(3.2%)		
Shape, n(%)	Regular	76(96.2%)	30(96.8%)	-	1.000
	Irregular	3(3.8%)	1(3.2%)		
CDFI, n(%)	0	8(10.1%)	9(29.0%)	10.583	0.014
	I	29(36.7%)	15(48.4%)		
	II	18(22.8%)	3(9.7%)		
	III	24(30.4%)	4(12.9%)		
Calcification, n(%)	Yes	75(94.9%)	30(96.8%)	-	0.679
	No	4(5.1%)	1(3.2%)		
Tumor diameter(cm): mean ± STD		4.50 ± 2.39	4.33 ± 2.19	0.340	0.735

### Comparison of qualitative and quantitative analysis of H-CEUS features between the CCRCC group and the AML group

The qualitative analysis of H-CEUS revealed significant differences between the two groups in terms of enhancement mode, regression mode, peak intensity, enhancement uniformity, absence of enhancement, and the presence or absence of a pseudocapsule (χ² = 41.614, -, -, = 2.758, = 42.099, -; *P* < 0.001, < 0.001, < 0.001, 0.097, < 0.001, and < 0.001, respectively). The H-CEUS imaging of CCRCC was mainly characterized by fast forward enhancement (56/79, 70.9%), slow regression (46/79, 58.2%), heterogeneity (65/79, 82.3%), and high enhancement (58/79, 73.4%). Among all cases, 65 (82.3%) showed no enhancement, whereas 67 (84.8%) had pseudocapsules. The H-CEUS imaging of AML cases primarily showed slow forward enhancement (26/31, 83.9%), slow regression (31/31, 100.0%), heterogeneity (21/31, 67.7%), and low enhancement (25/31, 80.6%). Only 5 cases (16.1%) had no enhanced areas and 1 case (3.2%) had a pseudocapsule. No statistically significant differences were observed in the enhancement direction, boundary after enhancement, and enhancement range between the two groups (all *P* > 0.05) (Table 2).

**Fig. 1:**
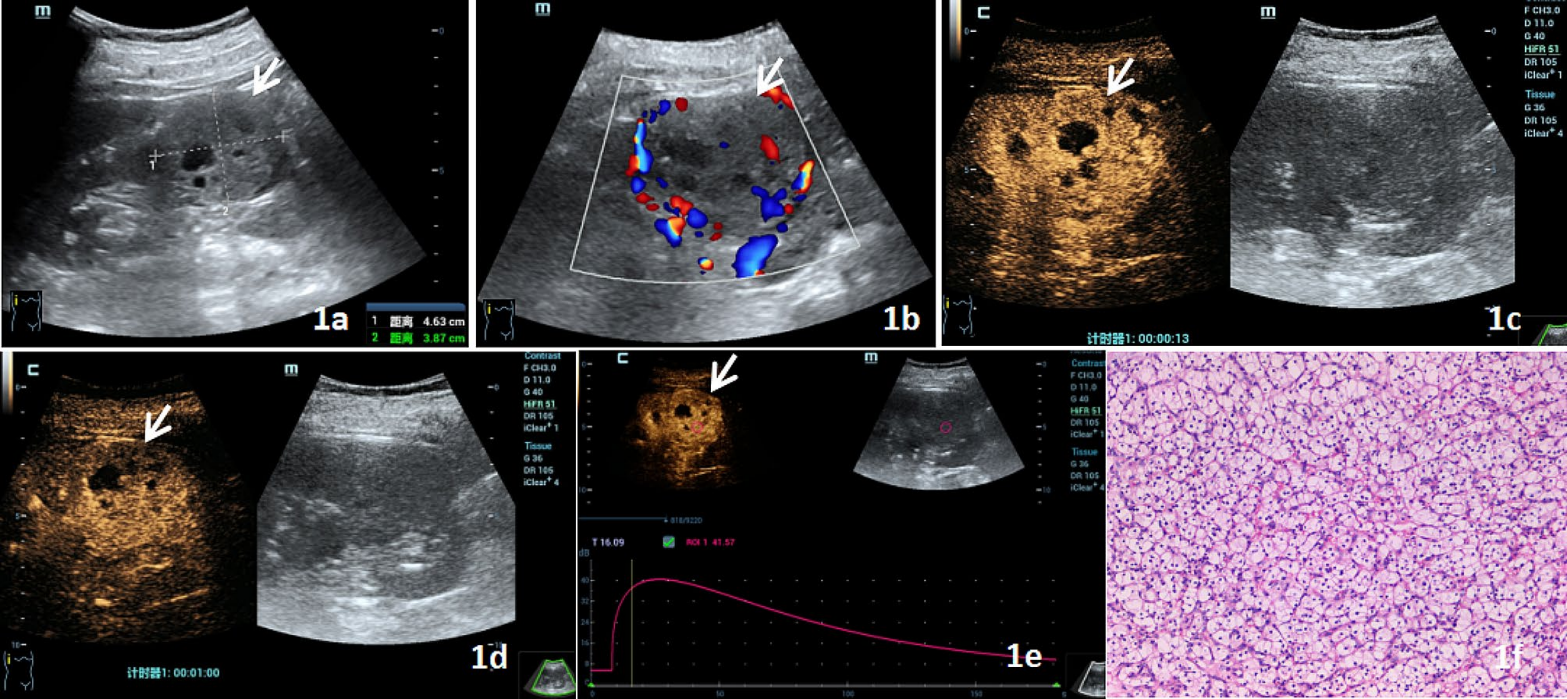
CCRCC US and H-CEUS images. Figure 1**a** shows a hypoechoic mass in the upper pole of the right kidney on US, with clear boundaries, regular shape, and visible anechoic areas. Figure 1**b** shows a relatively rich blood flow signal in the lesion, grade III, on CDFI. Figure 1**c** shows high enhancement in the 13 S perfusion period of H-CEUS. Figure 1**d** illustrates the 60s regression period, with the lesion showing slightly higher enhancement, fast forward and slow regression, and high enhancement of inhomogeneity, no enhancement in the interior, and annular enhancement in the periphery. Figure 1**e** displays the time-intensity curve and TIC parameters of the ROI of the lesion. Figure 1**f** shows the lesion was pathologically identified as renal clear cell carcinoma. When studied under the microscope following HE staining, the tumor cells seemed nested, with tiny and spherical nuclei, and empty and bright cytoplasm. Additionally, the distribution of thin-walled blood veins was noted (x 100). (Arrow indicates the location of the lesion)

**Fig. 2:**
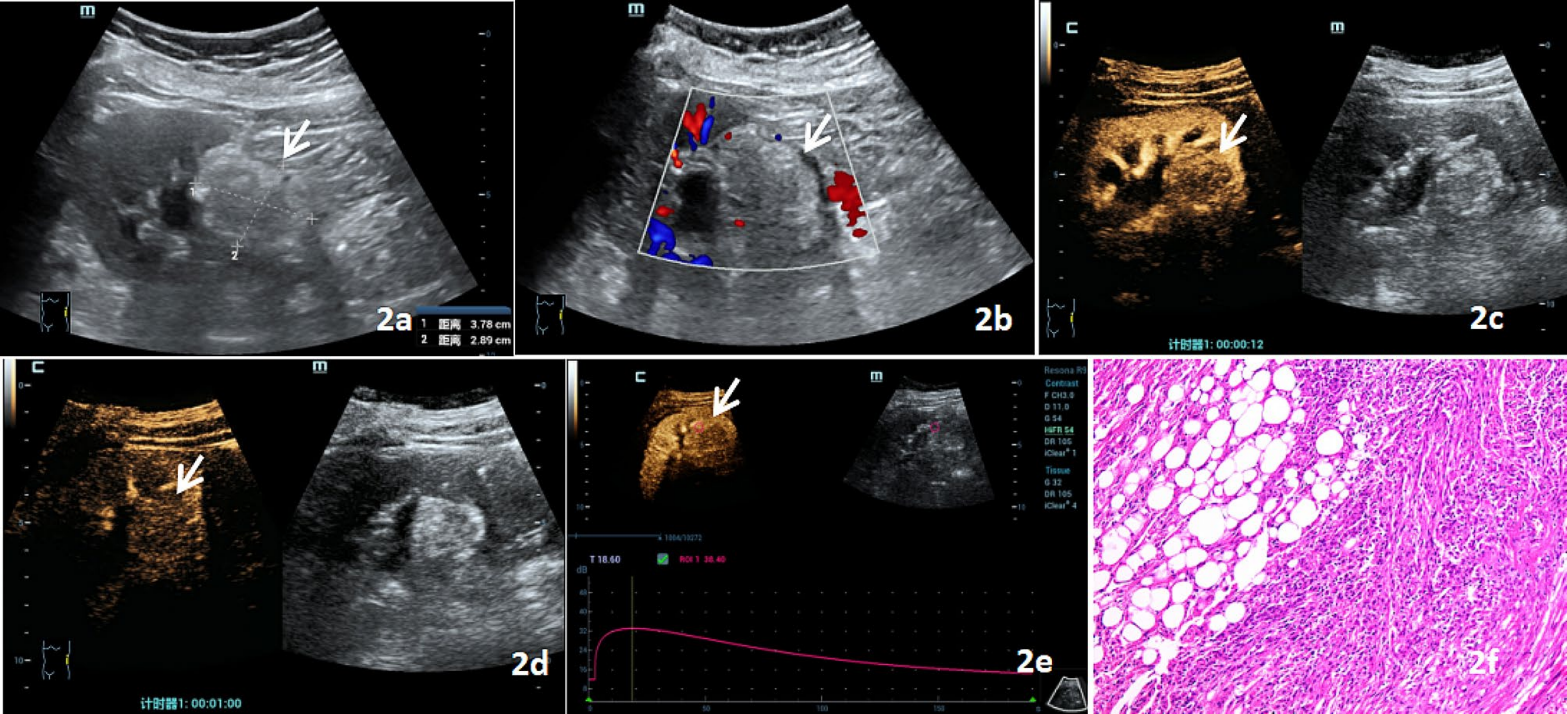
AML US and H-CEUS images. Figure 2**a** shows a hyperechoic mass in the lower pole of the left kidney on US, with clear boundaries and a regular shape. Figure 2**b** shows that punctate blood flow signals in the lesion on CDFI, grade I. Figure 2**c** shows low enhancement in the 13 S perfusion period of H-CEUS. Figure 2**d** illustrates the 60s regression period, with the lesion showing high enhancement, slow forward and slow regression, low enhancement of inhomogeneity, no enhancement in the interior, and no annular enhancement in the periphery. Figure 2**e** displays the time-intensity curve and TIC parameters of the ROI of the lesion. Figure 1**f** shows the lesion was pathologically diagnosed as renal AML. Under the microscope, most of the lesions were spindle-shaped cells with hyperplasia, which were arranged in bundles or disordered, and included thick-walled blood vessels and adipocytes. (HE staining ×100). (Arrow shows lesion)

Furthermore, the AT (arrival time) in the CCRCC group was significantly shorter than in the AML group (Z = -3.266, *P* = 0.001), as determined by the H-CEUS quantitative analysis of the TIC curve parameters in the ROI area of the lesions between the two groups. The CCRCC group exhibited significantly greater values for PI (peak intensity), AS (ascent slope), and AUC compared to the AML group (Z = -2.043, = -2.545, = -3.565; *P* = 0.041, = 0.011, and < 0.001, respectively). However, the differences in TTP (time to peak), DT/2 (1/2 descending time), DS (descending slope of curve), and MTT (mean transit time) between the two groups were not statistically.

significant (all *P* > 0.05) (Table 2).Representative examples of US, H-CEUS and pathological findings for CCRCC and AML are presented in Figs. 1 and 2.

**Table 2:** Qualitative and Quantitative analysis the features of H-CEUS in CCRCC and AML groups

		CCRCC(*n* = 79)	AML(*n* = 30)	χ² or Z	*p* Value
Enhancement mode	Earlier	56(70.9%)	1(3.2%)	*χ²=*41.614	< 0.001
	Equal	5(6.3%)	4(12.9%)		
	Slower	18(22.8%)	26(83.9%)		
Regression mode	Faster	33(41.8%)	0(0.0%)	-	< 0.001
	Equal	0(0.0%)	0(0.0%)		
	Slower	46(58.2%)	31(100.0%)		
Peak intensity	Low	20(25.3%)	25(80.6%)	-	< 0.001
	Equal	1(1.3%)	0(0.0%)		
	High	58(73.4%)	6(19.4%)		
Homogeneity	Homogeneous	14(17.7%)	10(32.3%)	*χ²=*2.758	0.097
	Heterogeneous	65(82.3%)	21(67.7%)		
No enhancement	Yes	65(82.3%)	5(16.1%)	*χ²=*42.099	< 0.001
	No	14(17.7%)	26(83.9%)		
Fill-in direction	Centripetal	58(73.4%)	23(74.2%)	-	1.000
	Entirety	20(25.3%)	8(25.8%)		
	Centrifugal	1(1.3%)	0(0.0%)		
Boundary after enhancement	Clear	78(98.7%)	31(100.0%)	*χ²=*0.396	0.529
	Unclear	1(1.3%)	0(0.0%)		
Range of enhancement	Enlarged	78(98.7%)	31(100.0%)	-	1.000
	Unenlarged	1(1.3%)	0(0.0%)		
Pseudocapsule	Yes	67(84.8%)	1(3.2%)	-	< 0.001
	No	12(15.2%)	30(96.8%)		
TIC	AT	6.21(4.88,9.10)	9.19(7.08,10.91)	*Z*=-3.266	0.001
	TTP	25.54(17.42,31.63)	28.33(22.98,34.09)	*Z*=-1.957	0.050
	PI	38.63(30.25,44.93)	32.92(28.69,41.18)	*Z*=-2.043	0.041
	AS	0.99(0.76,1.36)	0.73(0.59,1.10)	*Z*=-2.545	0.011
	DT/2	99.07(68.38,133.41)	111.88(86.31,135.70)	*Z*=-1.505	0.132
	DS	-0.17(-0.22,-0.13)	-0.15(-0.21,-0.11)	*Z*=-1.204	0.229
	AUC	2822.79(2104.52,3976.59)	1591.25(1146.64,2928.85)	*Z*=-3.565	< 0.001
	MTT	99.40(77.79,129.10)	93.7(61.87,124.51)	*Z*=-1.139	0.255

CCRCC, clear cell renal cell carcinoma; AML, angiomyolipoma; AT, arrival time; TTP, time to peak; PI, peak intensity; AS, ascent slope; DT/2, 1/2 descending time; DS, descending slope of curve; AUC, area under the curve; MTT, mean transit time

### Diagnostic efficacy of H-CEUS

The parameters of the clinical features, US, and H-CEUS features that exhibited statistically significant differences between the two groups were selected for logistics regression analysis, revealing that gender, nodule echo, and H-CEUS’s pseudocapsule, AS, and AUC were independent risk factors for CCRCC.(Table 3). Further analysis of the ROC curve indicated that the combination of gender and nodule echo exhibited a sensitivity of 92.4%, specificity of 64.5%, and an AUC of 0.847 in distinguishing between CCRCC and AML. The combination of the H-CEUS’s pseudocapsule, AS, and AUC for the differential diagnosis of CCRCC and AML showed an AUC of 0.918, a sensitivity of 84.8%, and a specificity of 96.8%. While the diagnostic efficacy of both methods showed no significant difference (Z = -1.286, *P* = 0.198), H-CEUS exhibited superior AUC and specificity.

**Table 3:** Binary logistics regression analysis of clinical, US and H-CEUS parameters for CCRCC

Features			OR(95CI)	*p* Value
Clinical + US	Gender	Male	1(reference)	
		Female	0.144(0.045,0.460)	0.001
	Echogenicity	Hypo-	1(reference)	
		Iso-	-	0.999
		Hyper-	0.097(0.028,0.338)	0.001
	CDFI	0	1(reference)	
		I	1.766(0.397,7.848)	0.455
		II	5.535(0.809,37.874)	0.081
		III	3.172(0.541,18.605)	0.201
Qualitative H-CEUS	Enhancement mode	Earlier	0.337(0.010,10.842)	0.539
		Equal	0.236(0.008,7.199)	0.408
		Slower	1(reference)	
	Regression mode	Faster	-	0.997
		Equal	-	-
		Slower	1(reference)	
	Peak intensity	High	1(reference)	
		Equal	-	1.000
		Low	3.219(0.114,90.812)	0.493
	No enhancement	Yes	3.465(0.372,32.32)	0.275
		No	1(reference)	
	Pseudocapsule	Yes	56.305(5.454,581.253)	0.001
		No	1(reference)	
Quantitative H-CEUS	TIC	AT	1.116(0.980,1.270)	0.097
		TTP	1.038(0.972,1.108)	0.266
		PI	0.961(0.886,1.042)	0.335
		AS	6.175(1.058,36.030)	0.043
		AUC	1.001(1.000,1.001)	0.013

AT, arrival time; TTP, time to peak; PI, peak intensity; AS, ascent slope; AUC, area under the curve

## Discussions

Prior studies suggested [14] a significant imbalance in the incidence of CCRCC between different genders. The incidence ratio between males and females is around 2:1. This may potentially be attributed to variations in the mutational spectra seen in tumors based on patient gender, such as mutations in X chromosome-encoded genes being more common in tumors derived from male patients, while BAP1 mutations are more commonly observed in tumors in female patients. Fittschen Astrid et al. conducted a retrospective analysis of abdominal ultrasound results of 61,389 patients [15], revealing that among 270 cases of sporadic AML, females showed a higher occurrence than males (2:1); the peak age of AML onset was between 40 and 60 years old. Furthermore, this study found a significant difference in gender between the CCRCC and AML groups. 62.0% of CCRCC patients were male, while 74.2% of AML patients were female. In addition, gender was also identified as an independent variable in subsequent regression analyses. In this study, statistically significant differences in surgical methods were observed between the CCRCC group and the AML group (all *P* < 0.05). In the AML group, 61.3% (19/31) of patients underwent partial nephrectomy and no patient underwent radical nephrectomy. In contrast, all patients in the CCRCC group underwent surgical treatment. Partial nephrectomy was performed in 19 cases of AML, of which 3 cases were epithelioid AML, 7 cases were anadipotic AML, and 9 cases were AML (diameter > 5 cm). Therefore, promptly and precisely distinguishing between CCRCC and AML may prevent unnecessary surgical intervention.

Ultrasound technology provides a rapid, secure, reliable, and cost-effective approach to identifying the fundamental features of the lesion, including its location, size, morphology, boundary, echoes, and blood supply. This study revealed significant differences in the US features, such as the echo of the mass and blood flow between the CCRCC group and the AML group (*P* < 0.05). US of AML showed a hyperechoic mass (83.9%) and inadequate blood flow in the nodule (77.4% of grade 0 and I). In contrast, the US of CCRCC was characterized by hypoechoic (68.4%) and abundant blood flow (53.2% of grade II and III blood flow). Notably, US can differentiate between benign and malignant renal lesions to a certain extent by examining the parameters of nodule echo and nodule blood flow. However, existing research [16] demonstrated that 30-60% of small renal carcinomas show a hyperechoic lesion on US, which cannot be distinguished from the hyperechoic lesion observed in AML. In addition, many malignant tumors do not possess the distinctive features of high-velocity blood flow. Although blood flow is abundant in malignant tumors, such lesions typically manifest as low-velocity blood flow. The current limitations of conventional color ultrasound include its inability to depict low-velocity blood flow within the tumor and blood flow in deeper or smaller tumors due to the instrument conditions and tumor location. Therefore, it is difficult to distinguish CCRCC from AML based on nodular echo and blood flow using conventional ultrasound, making it challenging to make an accurate diagnosis before surgery.

CEUS can accurately and sensitively assess the state of blood perfusion in the microcirculation and provides real-time and dynamic information on the microvascular perfusion of tissues. The findings improve the ability to detect the lesions and differentiate between benign and malignant lesions [17]. However, the rapid perfusion of renal lesions during conventional CEUS restricts the ability to acquire blood flow signals during the arterial phase, which poses certain limitations on the diagnosis of the disease. H-CEUS is a technique that enhances the temporal resolution of images by increasing the acquisition frame rate. The technique provides a greater temporal and spatial correlation resolution for evaluating vascular enhancement, specifically in microvessels [18]. Xiang Fei et al. [19] verified that H-CEUS enhanced temporal resolution by increasing the frame rate, which was beneficial for accurately depicting the differences in microcirculation in gallbladder polypoid lesions and enhancing the ability to differentiate between cholesterol polypoid lesions and adenoma. F Giangregorio et al. [20] discovered that HiFR-CEUS demonstrated greater vascularization of focal liver lesions (FLL) in the context of liver cirrhosis in the arterial phase compared to C-CEUS. The improved temporal resolution enabled more precise measurement of the perfusion details in the arterial phase.

This study conducted a qualitative and quantitative analysis of the features of H-CEUS in two groups of patients. A comparison of the two groups of H-CEUS revealed statistically significant distinctions in the following parameters: enhancement mode, regression mode, peak intensity, enhancement uniformity, no enhancement, and the presence or absence of a pseudocapsule. H-CEUS in CCRCC was characterized by fast forward enhancement (56/79, 70.9%), slow regression (46/79, 58.2%), heterogeneity (65/79, 82.3%), high enhancement (58/79, 73.4%), accompanied by non-enhanced area (82.3%), and pseudocapsule (84.8%). In contrast, the H-CEUS of AML was characterized by slow forward enhancement (26/31, 83.9%), slow regression (31/31, 100.0%), heterogeneity (21/31, 67.7%), low enhancement (25/31, 80.6%), and often without non-enhanced area and pseudocapsule. The discrepancy in the imaging findings may be attributed to the rich blood supply of CCRCC. Pathologically, CCRCC is characterized by the presence of transparent tumor cells and a dense network of thin-walled capillaries in the stroma. The internal vessel density is elevated, mostly exhibiting a dendritic structure with a larger vessel diameter. The tumor often exhibits cystic cavities, necrosis, and hemorrhage foci. Therefore, H-CEUS showed a fast flow-in with high enhancement of heterogeneity. The large number of arteriovenous fistulas in the tumor promotes the rapid clearance of the contrast agent, showing rapid regression. In contrast, AML lesions have low vascular components, thin lumens, and no elastic layer in the walls, thereby displaying slow perfusion and low enhancement. Additionally, the repeated circulation of microbubbles in the complex microvascular network may contribute to the slow regression of the contrast agent [21, 22]. The H-CEUS mode increased the number of image acquisitions, and the frame rate was increased to 50–65 Hz of HFR CEUS, which greatly improves the temporal resolution, and increases the image information and the contrast process, with both CCRCC and AML showing uneven enhancement. Irrespective of tumor malignancy, failure to acquire adequate nutrients for its development will result in ischemic necrosis, leading to the heterogeneity enhancement of tumor.

The H-CEUS results revealed no statistically significant difference was found in terms of the absence of enhancement and the presence of pseudocapsule between CCRCC patients and the AML patients in this study. The majority of the H-CEUS images of CCRCC exhibited no regions of enhancement (82.3%) and pseudocapsules (84.8%). In contrast, only 16.1% of AML images showed areas without enhancement, and only one case (3.2%) displayed a pseudocapsule. The difference in imaging findings may be attributed to CCRCC being a malignant tumor composed of clear or eosinophilic tumor cells with a fine vascular network inside the tumor. Cystic cavities, necrosis, and hemorrhage foci are often seen inside the tumor, which are difficult to visualize with conventional ultrasound. However, CEUS increases the contrast between the tumor tissue and the cystic cavity, necrosis, and hemorrhage foci, clearly displaying small anechoic areas, and showing no enhancement [23]. The pseudocapsule is mostly composed of fibrous tissues and a portion of the normal renal parenchyma. US typically struggles to identify the pseudocapsule due to its similarity in echo to both the tumor and the surrounding normal renal tissue. H-CEUS can clearly show the pseudocapsule, which is surrounds the tumor in a circular shape, and the enhancement time is observed to occur earlier, and the regression time later compared to the renal cortex, showing a circular high enhancement. The findings of this study indicated that the H-CEUS in the CCRCC group exhibited a greater occurrence of areas without enhancement and pseudocapsule, which aligns with prior literature studies [24].

This study further analyzed the TIC parameters of the ROI area of the lesion under the H-CEUS between the CCRCC and AML groups. The results revealed that the TIC parameter AT was lower in the CCRCC group compared to the AML group, whereas the AS was significantly higher in the CCRCC group than in the AML group. The terms AT and AS refer to arrival time and ascent slope in the perfusion period during the contrast process. These terms indicate that CCRCC has a faster forward and a higher slope of increase compared to AML. CCRCC is a highly vascularized malignant tumor characterized by extensive angiogenesis, which may account for this difference [17]. The TIC parameters PI and AUC of CCRCC were also significantly greater than in the AML group. This may be attributable to the fact that CCRCC is a lesion with an abundant blood supply and a significant number of arteriovenous fistulas. During the arterial phase, the contrast agent enters the lesion rapidly via the arteriovenous fistula, leading to a rapid accumulation of a substantial quantity of the contrast agent in the lesion [25]. In addition, although H-CEUS features enhanced the temporal resolution, it does not cause too much damage to the contrast agent, especially the reduction of the absorbtion of the contrast agent during the arterial phase, which shows high enhancement [26]. AML is a benign tumor with fewer blood vessels. Moreover, the tumor contains malformed blood vessels, irregularly thickened vessel walls, and narrow lumens, resulting in slow circulation of the contrast agent within the tumor. This leads to a reduction in the TIC curve PI and AUC [27].

Logistic regression was employed in this investigation to ascertain the independent risk factors associated with CCRCC, including gender, nodule echo, and H-CEUS parameters (pseudocapsule, AS, and AUC). Additional ROC analysis revealed that H-CEUS had a superior AUC in distinguishing between CCRCC and AML compared to the combination of gender and nodular echo. Although no significant difference in diagnostic efficiency was observed between the two, the diagnostic sensitivity and specificity achieved 87.4% and 97.5%, respectively. H-CEUS achieved the best AUC in differentiating CCRCC and AML, with a value of 0.918. H-CEUS is an ultra-wide low focal length-based nonlinear imaging technique that combines the fundamental wave, secondary harmonic, and higher harmonic signals produced by the ultrasound contrast agent to increase the frame rate and more thoroughly observe the microcirculation perfusion process of the rich blood supply lesion, thereby improving the accuracy of the contrast imaging process. In order to differentiate between CCRCC and AML, the AUC and specificity of H-CEUS were found to be superior when compared to those achieved by combing gender and nodule echo characteristics. Therefore, increasing the frame rate of H-CEUS enables a more precise differentiation between CCRCC and AML based on the different microvascular pathological characteristics of the two tumors.

Nevertheless, the limitations of this study should be acknowledged. Firstly, the number of AML cases examined in this study was smaller than that of CCRCC, necessitating a more extensive sample size for further assessment. Secondly, a particular connection between tumor size and the process of CEUS perfusion was hypothesized. However, this study did not categorize the tumor size, tumor grade, and stage separately for the two tumors. Finally, some cases of AML were not pathologically diagnosed, which might introduce bias, and different types of AML have certain differences in angiography methods of CEUS. No subgroup analysis was conducted on AML, which requires a larger sample size in future studies. In addition, our study excluded patients with deep tumor location, obesity or poor ultrasound penetration, whose CEUS images could not be interpreted, which may lead to bias in case selection. The attenuation caused by high concentration of microbubbles in the superficial layer of the renal cortex affects the imaging and observation of the deep tissue of the kidney. The false appearance of perfusion reduction and the corresponding quantitative parameter error lead to the deviation of the results. Therefore, The results of our study are suitable for renal tumors with a shallow location and good H-CEUS images.when the location of renal tumor is deep or the image quality of H-CEUS is poor, CT or MRI are recommended.

In summary, H-CEUS has higher sensitivity and specificity in the differential diagnosis of CCRCC and AML by improving the temporal resolution. This technology enables a more precise ultrasound-based diagnosis for patients with kidney tumors, guiding individualized therapy.

## Data Availability

Data is available upon reasonable request.
